# FoxO3 increases miR-34a to cause palmitate-induced cholangiocyte lipoapoptosis

**DOI:** 10.1194/jlr.M071357

**Published:** 2017-04-28

**Authors:** Sathish Kumar Natarajan, Bailey A. Stringham, Ashley M. Mohr, Cody J. Wehrkamp, Sizhao Lu, Mary Anne Phillippi, Dee Harrison-Findik, Justin L. Mott

**Affiliations:** Department of Biochemistry and Molecular Biology, University of Nebraska Medical Center, Omaha NE

**Keywords:** nonalcoholic fatty liver disease, steatohepatitis, microRNA-34a, forkhead box transcription factor class O3

## Abstract

Nonalcoholic steatohepatitis (NASH) patients have elevated plasma saturated free fatty acid levels. These toxic fatty acids can induce liver cell death and our recent results demonstrated that the biliary epithelium may be susceptible to lipotoxicity. Here, we explored the molecular mechanisms of cholangiocyte lipoapoptosis in cell culture and in an animal model of NASH. Treatment of cholangiocytes with palmitate (PA) showed increased caspase 3/7 activity and increased levels of cleaved poly (ADP-ribose) polymerase and cleaved caspase 3, demonstrating cholangiocyte lipoapoptosis. Interestingly, treatment with PA significantly increased the levels of microRNA miR-34a, a pro-apoptotic microRNA known to be elevated in NASH. PA induction of miR-34a was abolished in cholangiocytes transduced with forkhead family of transcription factor class O (FoxO)3 shRNA, demonstrating that FoxO3 activation is upstream of miR-34a and suggesting that FoxO3 is a novel transcriptional regulator of miR-34a. Further, anti-miR-34a protected cholangiocytes from PA-induced lipoapoptosis. Direct and indirect targets of miR-34a, such as SIRT1, receptor tyrosine kinase (MET), Kruppel-like factor 4, fibroblast growth factor receptor (FGFR)1, and FGFR4, were all decreased in PA-treated cholangiocytes. SIRT1 and MET were partially rescued by a miR-34a antagonist. Cholangiocyte apoptosis and miR-34a were dramatically increased in the liver of mice with early histologic features of NASH. Our study provides evidence for the pro-apoptotic role of miR-34a in PA-induced cholangiocyte lipoapoptosis in culture and in the liver.

The incidence of nonalcoholic fatty liver disease (NAFLD) is skyrocketing in both developed and developing countries due, in large part, to the increased prevalence of obesity. NAFLD is the hepatic manifestation of metabolic syndrome and is accompanied by increased FFAs in circulation. These increased free fatty acids (FFAs) are due to excessive adipose tissue lipolysis and are toxic to cells in the liver, heart, and pancreas (1, 2). Hepatocyte lipoapoptosis due to increased circulating FFAs is recognized as a hallmark of nonalcoholic steatohepatitis (NASH), a severe form of NAFLD. In addition to hepatocyte injury, cholestatic NAFLD has been suggested to develop at least in a subset of patients with NAFLD (3). In support of this notion, we have recently demonstrated that biliary epithelial cells, or cholangiocytes, undergo lipoapoptosis upon saturated FFA exposure. Saturated FFAs triggered activation of stress kinases, like p38 and ERK. We also found that FFA-induced cholangiocyte lipoapoptosis was dependent on the forkhead family of transcription factor class O (FoxO)3, and, in part, its downstream target, p53-upregulated modulator of apoptosis (PUMA) (4, 5).

MicroRNAs are noncoding small RNAs that mediate posttranscriptional regulation of mRNA transcript levels and protein translation. They have been shown to play an important role in a variety of cellular processes, including apoptosis. In particular, miR-34a is a pro-apoptotic microRNA that decreases expression of several cell survival proteins (6–9). Additionally, miR-34a expression is induced by p53 at the promoter level to increase apoptosis (10, 11). miR-34a has been suggested to modulate apoptosis through the SIRT1/p53 pathway (9, 12, 13). NAFLD and NASH patients have elevated levels of pro-apoptotic miR-34a in the circulation and the liver. Increased miR-34a levels were also shown to be associated with the severity of NASH in patients (14, 15). However, the exact role for miR-34a in cholangiocyte lipoapoptosis is unknown.

In the present study, we hypothesized that miR-34a is important in cholangiocyte lipoapoptosis. We explored the critical role for miR-34a during cholangiocyte lipoapoptosis induced by palmitate (PA). Treatment of cholangiocytes with PA induced the expression of miR-34a, reduced expression of several miR-34a target proteins, and increased lipoapoptosis. We also identified miR-34a as a novel target of FoxO3.

## MATERIALS AND METHODS

### Materials

Palmitic acid (#P5585) and fatty acid-free BSA (#A3803) were obtained from Sigma-Aldrich, St. Louis, MO. Magnetic protein G beads (#S1430S) were purchased from New England Bio Labs, Ipswich, MA. Trizol reagent and all other chemicals were obtained from Thermo Fisher Scientific, Waltham, MA.

### Animals

All procedures involving animals in this work were approved by the IACUC at the University of Nebraska Medical Center. C57BL/6J mice (4–6 weeks old) were randomly divided into two groups and fed either control diet (17.2% kcal from fat, 100 g/kg sucrose) or high-fat high-sucrose (HFHS) diet (42% kcal from fat with 54% saturated and 9.7% trans-fat, 0.4% cholesterol, and 340 g/kg sucrose) for 3 months. Water was given ad libitum and the HFHS mice were fed sucrose-containing water (40 g/l) to mimic the Western diet, as described (16).

### Masson trichrome staining

Liver tissues were formalin-fixed, paraffin embedded, and stained for Masson trichrome reaction by the Tissue Sciences core facility at the University of Nebraska Medical Center.

### Antibodies

Rabbit antiserum against FoxO3 (2497), cleaved caspase 3 (C9661), and cleaved poly (ADP-ribose) polymerase **(**PARP) (P9542) were from Cell Signaling. Rabbit antiserum against PUMA (28226) was from Santa Cruz. Goat anti-lamin B (sc-6216) and mouse anti-actin (sc-1615) were purchased from Sigma. Peroxidase-conjugated secondary antisera were from Jackson Immuno Research laboratory.

### Cell lines and treatment

H69, a human normal immortalized cholangiocyte cell line, was grown in DMEM supplemented with 10% FBS, insulin (5 μg/ml), adenine (24.3 μg/ml), epinephrine (1 μg/ml), triiodothyronine-transferrin (triiodothyronine, 2.23 ng/ml; transferrin, 8.19 μg/ml), epidermal growth factor (9.9 ng/ml), and hydrocortisone (5.34 μg/ml). KMCH, Mz-ChA-1 and HuCCT-1 (human cholangiocarcinoma cell lines), MDA-KKA2 (mouse cholangiocyte cell line), and Huh7 (hepatoma cell line) were grown in DMEM supplemented with 10% FBS, insulin (0.5 μg/ml), and G418 (50 μg/ml) (5). H69, KMCH, HuCCT, Mz-ChA-1, Huh7, and MDAKK-2 cells were treated with the indicated concentrations of FFAs (400–800 μM) dissolved fresh in isopropanol at 100× and added to prewarmed medium containing 1% fatty acid-free BSA for 24 h. Vehicle treatment was isopropanol with a final concentration of 1% in the medium. The SIRT1 inhibitor, EX527, was used at 100 μM and the receptor tyrosine kinase (MET) inhibitor, Su11274, was used at 2 μM.

### Measurement of apoptosis

Percent apoptosis was quantified by characteristic nuclear morphology visualized by treatment with DNA-binding dye, DAPI, as described before (6, 7). Briefly, cells were stained with 5 μg/ml DAPI for 20–30 min at 37°C. Images were obtained under UV epifluorescence microscopy using a Leica DMI6000B inverted microscope. Apoptotic nuclei (condensed, fragmented) were counted and presented as a percent of total nuclei. At least 100 cells were counted per well and experiments were performed in triplicate. Caspase 3/7 activity was measured by enzymatic fluorophore release (Apo-ONE) according to the manufacturer’s instructions (Promega) and were represented as fold-change compared with vehicle treatment, with experiments performed in quadruplicate as described (5).

### Nuclear isolation and Western blot analysis

Nuclear extracts were prepared as described (17). Cell lysates containing 30 μg of protein were resolved by SDS-PAGE. Proteins were transferred to a nitrocellulose membrane and visualized by immunoblotting.

### Lentiviral shRNA transduction

shRNA silencing lentiviral pLKO.1-puro vector targeting FoxO3 and control GFP were obtained from Sigma and stable transfections were carried out as described (5). FoxO3 shRNA #1 and #2 target the nucleotide sequences 2185-2205 and 1626-1648 of FoxO3 mRNA (NM_001455.1), respectively.

### Isolation and quantitation of miR-34a

Cellular RNA and total liver RNA were isolated using lysis in chaotropic salt and acid:phenol:chloroform extraction (mirVana kit; Life Technologies). Total RNA (100 ng) was used to amplify miRs using hydrolysis probe and stem loop primers for miR-34a (hsa-miR-34a-5p, #PN4427975-000426) and Z30 control RNA (#PN4427975-001092). Relative expression was calculated using the delta CT method.

### miR-34a mimic and locked nucleic acid transfection

H69 and KMCH cells were transfected for 24 h with negative control A, a nontargeting control locked nucleic acid (LNA) oligonucleotide, or LNA-34a (250999-B) obtained from Exiqon. Cells were then treated with vehicle or PA (800 μM) for another 24 h and apoptotic markers were measured as described above. For miR-34a targets, cells were transfected with either miR-34a mimic (to replicate fatty acid-induced expression) or antagonist (LNA-34a) for 24 h followed by protein isolation.

### Statistics

Data are expressed as mean ± SEM. Statistical analysis was performed using one-way ANOVA with Bonferroni post hoc correction. *P* < 0.05 was considered to be statistically significant.

## RESULTS

### Evidence for PA-induced cholangiocyte lipoapoptosis

**Fig. 1.**
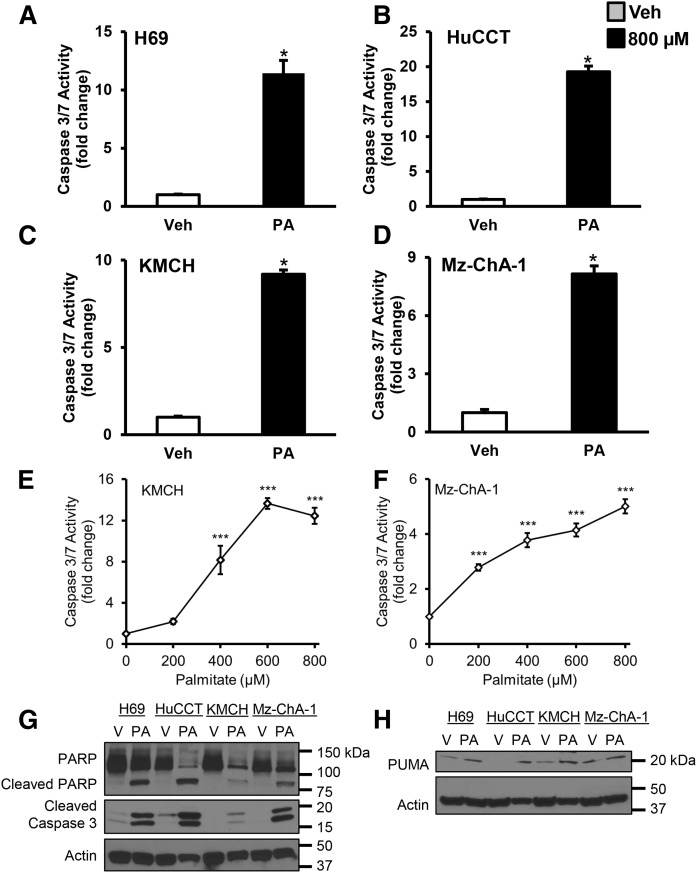
PA induced cholangiocyte lipoapoptosis. Cell lines were treated with PA (800 μM) or vehicle (Veh) for 24 h. A–D: Caspase 3/7 activity was significantly increased in PA-treated cells. Data represent the mean ± SEM for n = 4. *P < 0.05, compared with vehicle by *t*-test. KMCH cells (E) and Mz-ChA-1 cells (F) treated with PA (0–800 μM) showed a significant concentration-dependent increase in caspase 3/7 activity. Data represent the mean ± SEM for n = 4. ***P < 0.05, compared with vehicle; statistical comparison was by ANOVA with post hoc correction. G: Cleaved PARP and cleaved caspase 3 levels were increased in whole-cell lysates taken from PA-treated cells compared with vehicle-treated (V) cells. H: PUMA protein was also increased by PA treatment.

Our earlier report had established that cholangiocytes undergo lipoapoptosis upon FFA exposure (5). We treated normal immortalized and malignant cholangiocytes with PA (800 μM) for 24 h and measured caspase 3/7 activation. H69 cells showed a 10-fold increase in caspase 3/7 activity with PA treatment compared with vehicle (**Fig. 1A**). Cholangiocyte cell lines, HuCCT, KMCH, and Mz-ChA-1, showed a 20-fold, 9-fold, and 8-fold increase in caspase 3/7 activity, respectively, with PA treatment compared with vehicle (Fig. 1B–D). We tested PA concentrations from 0 to 800 μM and found increased apoptosis in KMCH cells over 400 μM (Fig. 1E) or over 200 μM in Mz-ChA-1 cells (Fig. 1F). In addition to caspase activation, we assessed the caspase cleavage products, PARP and caspase 3 itself. PARP is a 116 kDa protein with a role in DNA repair, chromatin structure formation, and differentiation. Increased caspase 3/7 activity with PA treatment was accompanied by an increase in the levels of 85 kDa cleaved PARP and cleaved caspase 3 in cholangiocytes (Fig. 1G). We have reported that the pro-apoptotic protein, PUMA, a downstream target of FoxO3 was induced by PA and was partly responsible for cholangiocyte lipoapoptosis (5). Here, we tested the expression of PUMA with PA treatment in different cholangiocyte cell lines and found that the expression was increased in H69, HuCCT, KMCH, and Mz-ChA-1 cells treated with PA compared with cells treated with vehicle (Fig. 1H). These results suggest that treatment with PA, comparable to pathophysiological levels found in NASH patients, induced cholangiocyte lipoapoptosis.

### PA induced miR-34a expression

**Fig. 2.**
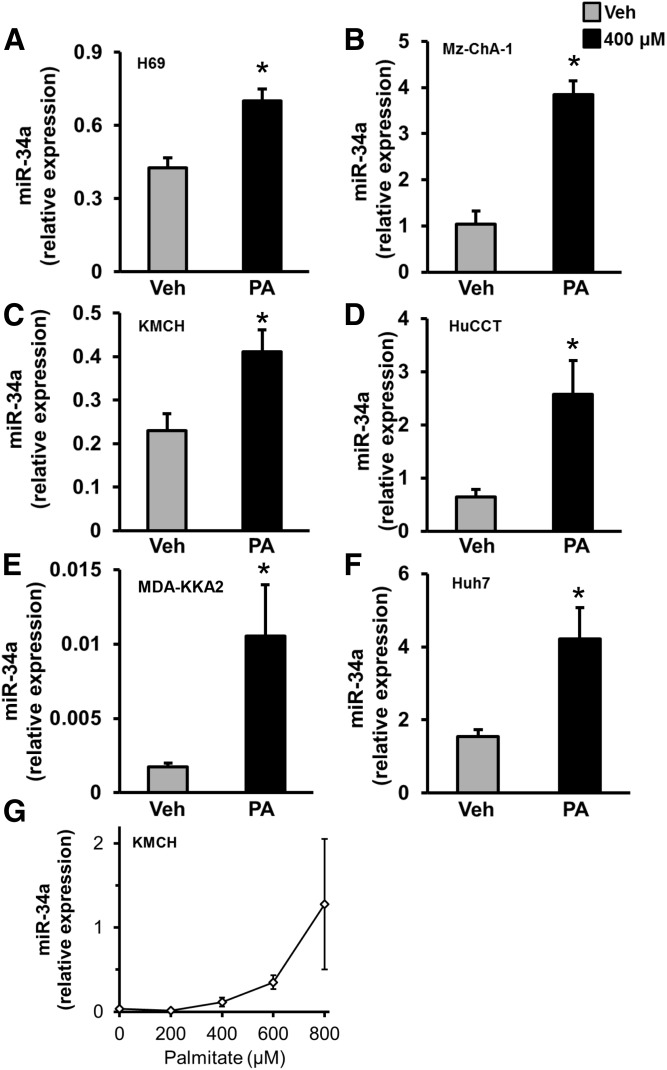
Induction of miR-34a by PA. A–F: Cell lines (cholangiocyte- or cholangiocarcinoma-derived: H69, Mz-ChA-1, KMCH, HuCCT, and MDA-KKA2; hepatoma-derived: Huh7) were treated with PA (400 μM) or vehicle (Veh) for 24 h. This lower concentration was chosen to limit cell death from PA allowing reliable RNA isolation. Relative expression of pro-apoptotic miR-34a was significantly elevated in PA-treated cholangiocytes and hepatocytes. miR-34a was quantified by RT-PCR and normalized to Z30 for human cell lines or RNU6 for mouse MDA-KKA2 cells. G: The full concentration range of PA from 200 to 800 μM was tested in KMCH cells and showed increased levels of miR-34a with an increasing concentration of PA. Data represent the mean ± SEM for n = 4. *P < 0.05, *t*-test.

To further evaluate the mechanism of cholangiocyte lipoapoptosis, we tested the role of the pro-apoptotic microRNA, miR-34a, in cholangiocyte lipoapoptosis. miR-34a was chosen because it was elevated in the liver and circulation from patients with NASH or diabetes (14, 15, 18). We analyzed miR-34a expression upon exposure to a lower concentration of PA (400 μM instead of 800 μM). We know that 400–800 μM PA induces a concentration-dependent increase in cell death (5), so initially we chose the lower concentration to minimize RNA loss from apoptosis. We found that miR-34a levels were significantly increased in human cholangiocyte cell lines, H69, Mz-ChA-1, KMCH, and HuCCT, treated with 400 μM PA (**Fig. 2A–D**). To test the generality of our finding, we used a mouse cholangiocyte cell line, MDA-KKA2. PA treatment showed an increase in miR-34a expression relative to vehicle-treated MDA-KKA2 cells (Fig. 2E). We next tested to determine whether PA also induced an increase in miR-34a expression in hepatocytes. A hepatoma cell line, Huh7, was treated with 400 μM PA for 24 h and miR-34a expression levels were measured. Similar to cholangiocytes, we observed that PA treatment induced expression of miR-34a in this hepatocyte-derived cell line (Fig. 2F). PA treatment of KMCH cells at 400, 600, or 800 μM progressively increased miR-34a levels (Fig. 2G). Together, these results suggest that PA induces the expression of miR-34a in both cholangiocytes and hepatocytes, possibly contributing to lipoapoptosis.

### Anti-miR-34a protects cholangiocyte lipoapoptosis

**Fig. 3.**
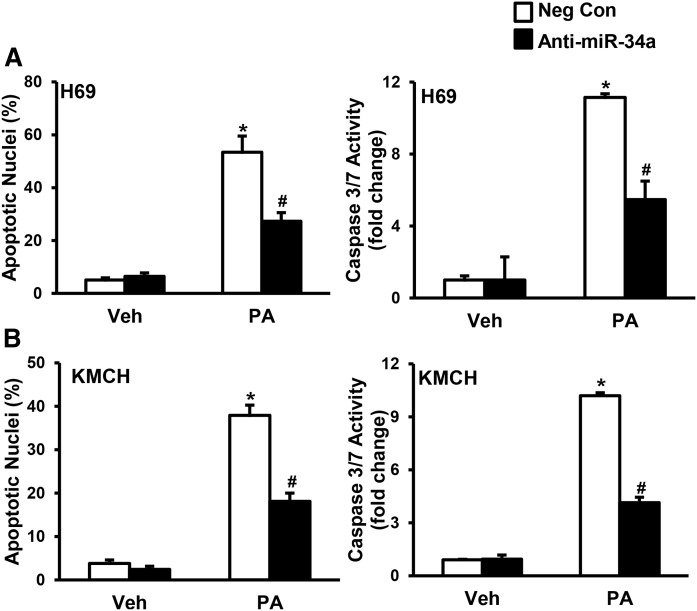
Anti-miR-34a protected against PA-induced cholangiocyte lipoapoptosis. A, B: miR-34a was antagonized by transfecting cells with LNA-34a (anti-miR-34a; filled bars) compared with nontargeting negative control A LNA (Neg Con; open bars). Twenty-four hours later, cells were treated with PA (800 μM) or vehicle (Veh) for 24 h. PA-induced cholangiocyte lipoapoptosis was measured by nuclear morphology (left) and by caspase 3/7 activity (right). Data represent the mean ± SEM for n = 4. *P < 0.001, compared with vehicle; #*P* < 0.001, compared with Neg Con with PA treatment; statistical comparison was by ANOVA with post hoc correction.

To test the role of miR-34a in cholangiocyte lipoapoptosis, we transfected H69 and KMCH cells with antisense LNA against miR-34a (anti-miR-34a) or negative control before challenging with PA. Treatment with PA induced cholangiocyte lipoapoptosis in H69 and KMCH cells transfected with negative control, as evidenced by an increase in percent apoptotic nuclei and caspase 3/7 activity (**Fig. 3**). H69 cells transfected with anti-miR-34a showed a significant decrease in percent apoptotic nuclei and caspase 3/7 activity with PA treatment (Fig. 3A). Similarly, anti-miR-34a transfected KMCH cells showed a significant decrease in PA-induced cholangiocyte lipoapoptosis (Fig. 3B). These results suggest an important role for miR-34a in PA-induced cholangiocyte lipoapoptosis.

### FoxO3 is critical for miR-34a expression

**Fig. 4.**
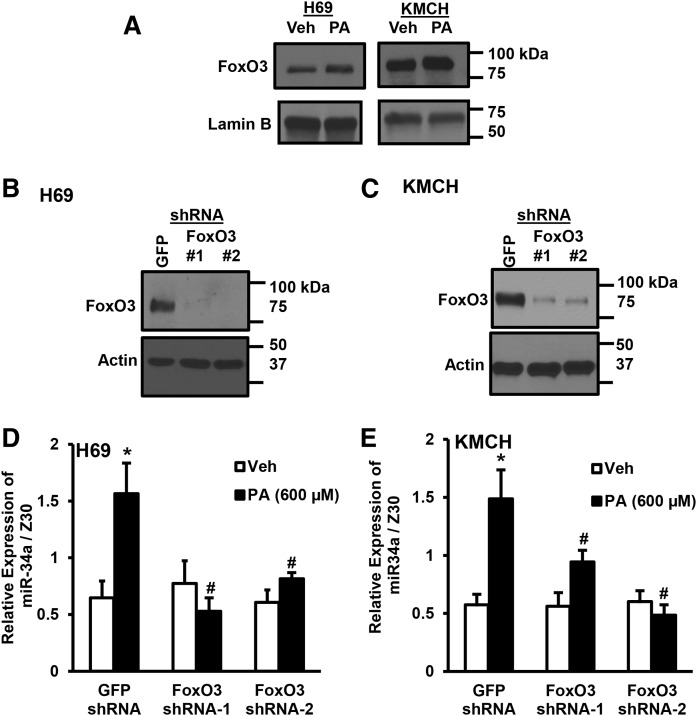
FoxO3 induced miR-34a expression. A: Nuclear extracts were prepared from H69 or KMCH cells treated with vehicle (Veh) or PA for 24 h. FoxO3 and lamin B were detected by immunoblot. B, C: Cell lines (H69 or KMCH) were transduced with shRNA to GFP or FoxO3 (two shRNAs, indicated as #1 or #2) and stably-transfected cells selected by antibiotic resistance. Whole-cell FoxO3 protein levels were compared with actin. D, E: GFP shRNA- or FoxO3 shRNA-transduced cells were treated with PA (600 μM; filled bars) or vehicle (Veh; open bars) for 24 h. miR-34a levels were normalized to Z30. Data represent the mean ± SEM for n = 4. *P < 0.05, compared with vehicle; #*P* < 0.05 compared with PA-treated GFP shRNA cells; ANOVA with post hoc correction.

Our earlier report demonstrated a critical role of FoxO3 in cholangiocyte lipoapoptosis (5). Here, we tested to determine whether FoxO3 is required for the induction of miR-34a during PA lipotoxicity. PA-treated H69 and KMCH cells showed an increase in nuclear FoxO3 levels after 16 h (**Fig. 4A**). Transduction with FoxO3 shRNA in H69 and KMCH cells resulted in knockdown of FoxO3 protein, as compared with control GFP shRNA transduced cells (Fig. 4B, C). Control shRNA- and FoxO3 shRNA-transduced cells were treated with 600 μM PA or vehicle for 24 h and miR-34a levels were analyzed by quantitative RT-PCR. PA significantly induced the expression of miR-34a in H69 cells transduced with control shRNA. However, increased miR-34a expression due to PA treatment was abolished in FoxO3 shRNA-transduced cells (Fig. 4D). Similarly, the induction of miR-34a by PA was significantly abrogated in KMCH cells transduced with FoxO3 shRNA (Fig. 4E). These data suggest that FoxO3 is a novel transcriptional regulator of miR-34a.

### miR-34a target expression

**Fig. 5.**
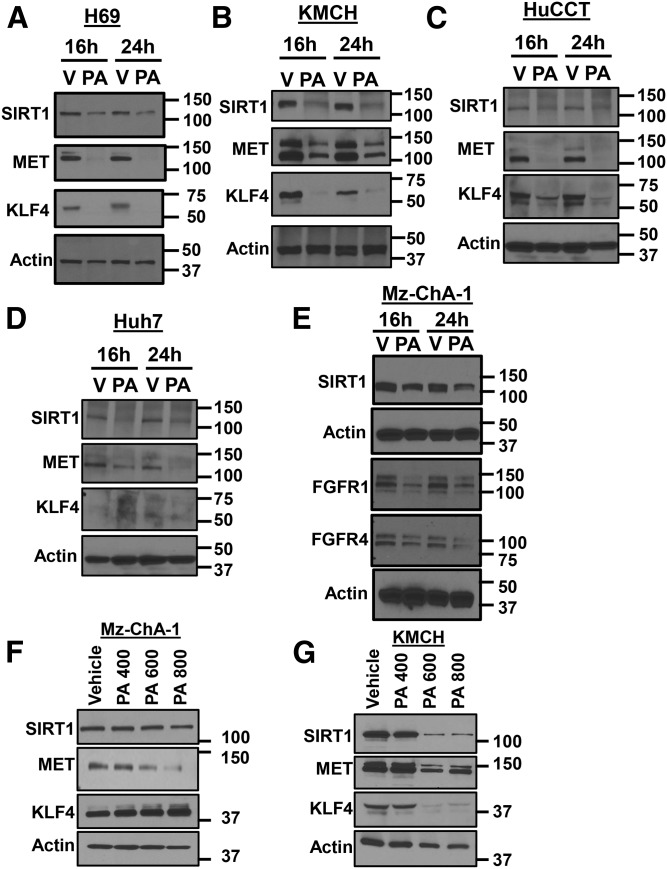
PA decreased SIRT1, MET, and KLF4. A–D: Cells were treated with PA (800 μM) or vehicle (V) for 16 or 24 h. SIRT1, MET, and KLF4 protein expression was detected by immunoblot, compared with actin control. E: Cells were treated as in (A–D) and SIRT1, FGFR1, or FGFR4 was detected by immunoblot compared with actin control. F, G: Increasing PA (400, 600, or 800 μM) was added to Mz-ChA-1 or KMCH cells and SIRT1, MET, and KLF4 proteins were measured by immunoblot.

The mRNAs of *SIRT1*, *MET*, and Kruppel-like factor 4 (*KLF4*) comprise of noncanonical and canonical seed binding sites for miR-34a (6, 9, 12, 13, 19–22). We then assessed expression levels of these anti-apoptotic proteins after 16 or 24 h of PA treatment. The levels of SIRT1 in H69 cells were found to be decreased after 16 or 24 h with 800 μM PA treatment compared with vehicle (**Fig. 5A**). H69 cells also showed a decrease in MET expression after 16 and 24 h of PA treatment (Fig. 5A). Levels of KLF4 were also lower in H69 cells treated with PA. Similarly, KMCH and HuCCT cells showed a decrease in the expression of SIRT1, MET, and KLF4 with 800 μM of PA treatment at the 16 and 24 h time points (Fig. 5B, C). We next tested to determine whether this was also true for the Huh7 hepatocyte cell line. Treatment with 800 μM PA led to a decrease in the expression of SIRT1 and MET, both after the 16 and 24 h time points. We did not find any expression of KLF4 in Huh7 cells and the absence of KLF4 was previously observed in proteomic studies of Huh7 cells (23, 24).

Levels of SIRT1 were decreased in Mz-ChA-1 cells after 16 or 24 h of PA treatment (Fig. 5E). β-Klotho and fibroblast growth factor receptor (FGFR)1 have been shown to be targets of miR-34a (25). Further, FGFR1 and FGFR4 are dependent on the coreceptor, β-klotho (26). Both FGFR1 and FGFR4 levels were decreased in cells exposed to PA after 16 or 24 h of treatment (Fig. 5E). We were unable to identify a sensitive and specific antibody to detect β-klotho protein.

Increasing PA concentrations decreased the protein levels of SIRT1, MET, and KLF4. In Mz-ChA-1 cells, SIRT1 did not show large expression changes (Fig. 5F), while MET was progressively decreased with increasing the concentration of PA (Fig. 5F). In KMCH cells, increasing PA decreased SIRT1, MET, and KLF4, especially at 600–800 μM (Fig. 5G). For Fig. 5F, G, the caspase inhibitor ZVAD-FMK was included (10 μM) to limit loss of cells to lipoapoptosis. The decrease in multiple cellular proteins was specific, as we did not observe a decrease in actin, and observed an increase in PUMA levels upon PA treatment. These results suggest that miR-34a upregulation with PA treatment has many cellular targets and decreases the levels of cell survival proteins, like SIRT1, MET, KLF4, FGFR1, and FGFR4, potentially contributing to cholangiocyte lipoapoptosis.

### miR-34a target validation

**Fig. 6.**
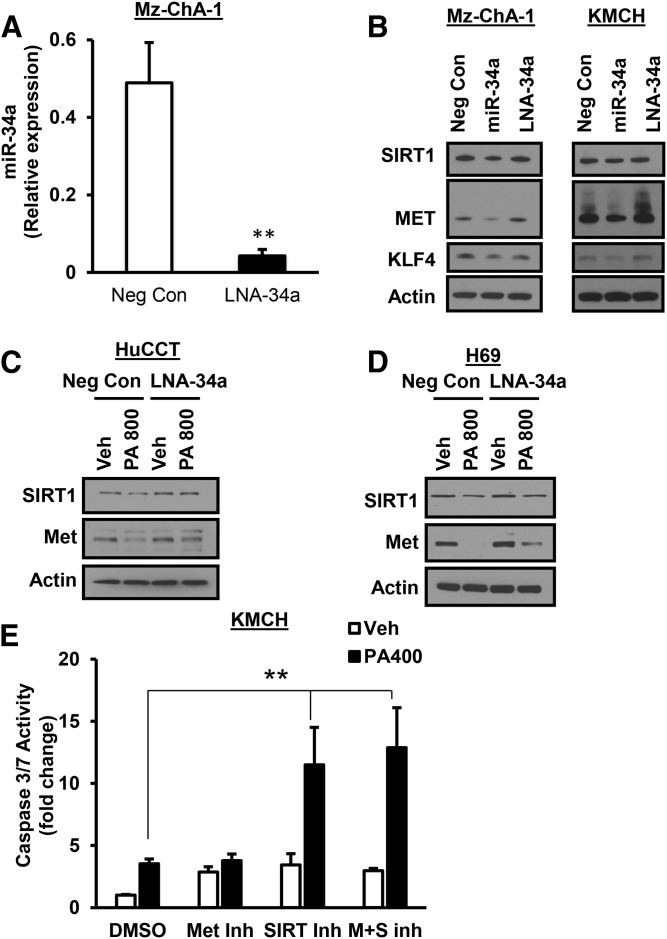
miR-34a decreased SIRT1, MET, and KLF4 protein expression. A: LNA-34a decreased miR-34a levels by 91% in Mz-ChA-1 cells 24 h after transfection. B: Mz-ChA-1 or KMCH cells were transfected with negative control A LNA (Neg Con), microRNA mimic (miR-34a), or anti-miR-34a (LNA-34a) for 24 h. SIRT1, MET, and KLF4 expression was detected by immunoblot in whole-cell lysates. C, D: SIRT1 and MET loss in response to PA was measured in cells transfected with negative control A LNA (Neg Con) or miR-34a antagonist (LNA-34a). E: Lipoapoptosis in response to a low PA stimulus was tested in the presence of DMSO vehicle, Su11274 MET inhibitor (2 μM), EX527 SIRT1 inhibitor (100 μM), or both inhibitors. Apoptosis was assessed by caspase 3/7 activity. **P < 0.01; *t*-test in (A), ANOVA with Bonferroni post hoc correction in (E).

We next wanted to further validate miR-34a targets by transfecting miR-34a mimic and anti-miR-34a in Mz-ChA-1 and KMCH cells. The miR-34a LNA antagonist decreased miR-34a expression by approximately 91% in Mz-ChA-1 cells (**Fig. 6A**). The miR-34a mimic decreased the expression of SIRT1, MET, and KLF4 in Mz-ChA-1 cells compared to negative-control transfected cells (Fig. 6B). Cells transfected with anti-miR-34a did not have dramatically altered levels of these proteins (Fig. 6B). KMCH cells transfected with miR-34a mimic showed a similar decrease in SIRT1, MET, and KLF4 (Fig. 6B). When cells were transfected with LNA-34a followed by PA treatment (800 μM), the miR-34a antagonist partially prevented loss of SIRT1 and MET (Fig. 6C, D). To test whether loss of function of either of these proteins contributes to lipoapoptosis, we treated KMCH cells with the SIRT1 inhibitor, EX527, or the MET kinase inhibitor, Su11274, followed by 400 μM PA. The lower PA concentration was chosen to allow for detection of sensitization to PA. The SIRT1 inhibitor, either alone or in combination, increased lipoapoptosis, while the MET inhibitor did not alter caspase 3/7 activation (Fig. 6E). Together, cells transfected with miR-34a mimic to increase miR-34a in the absence of PA treatment showed a decrease in predicted target proteins, such as SIRT1, MET, and KLF4. Antagonism of miR-34a partially protected from this loss, while inhibition of SIRT1 plus PA treatment exacerbated cell death.

### Cholangiocyte lipoapoptosis in HFHS-fed mice

**Fig. 7.**
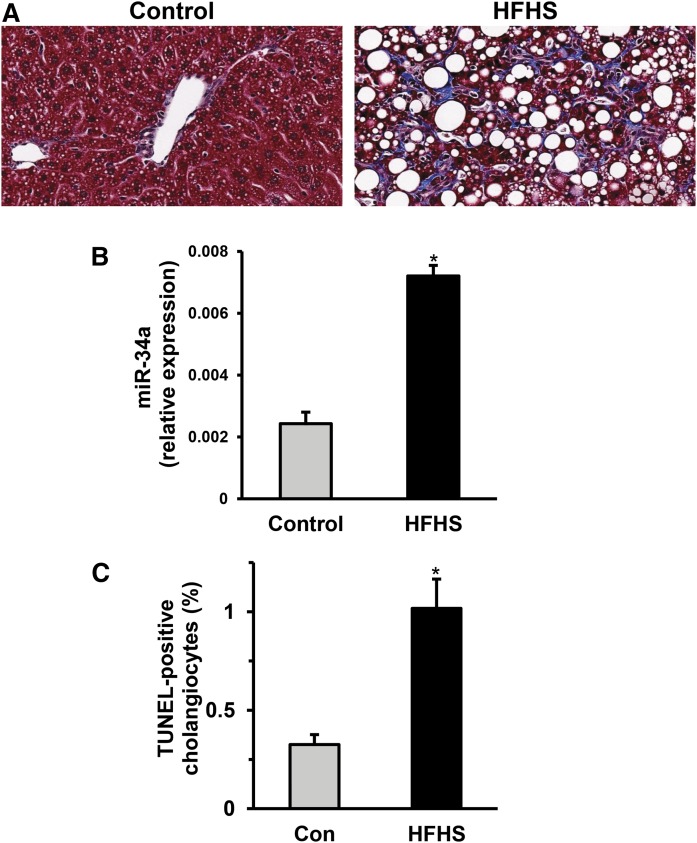
Cholangiocyte lipoapoptosis in animal model of NASH. A: A Masson trichrome-stained liver section from HFHS-fed mice showed enhanced lipid droplet and collagen accumulation compared with chow-fed control (Con) mice. B: Total RNA was isolated from flash-frozen liver tissue and miR-34a levels were measured relative to RNU6. C: Cholangiocytes were identified by pan-keratin staining and apoptotic nuclei were identified in the same section by TUNEL reaction. TUNEL-positive cholangiocytes are indicated as a percentage of total cholangiocyte nuclei. A total of 900–3,000 cholangiocytes were scored per sample. Data represent the mean ± SEM for n = 4 mice for each group. *P < 0.05, *t*-test.

C57BL/6 mice were fed with control or HFHS diet for 3 months. Liver sections were analyzed with Masson trichrome staining for the detection of chicken-wire-like collagen accumulation. Liver sections obtained from HFHS-fed mice showed lipid droplet and collagen accumulation compared with control-fed mice (**Fig. 7A**). We isolated liver total RNA and found that miR-34a levels were significantly upregulated in HFHS diet-fed mice compared with controls (Fig. 7B). Next, we co-stained formalin-fixed liver sections with pan-keratin (a bile duct marker) and TUNEL for the quantitation of apoptotic cholangiocytes. We observed elevated cholangiocyte lipoapoptosis in HFHS-fed mice (Fig. 7C). Our data provide evidence for the upregulation of hepatic miR-34a and for cholangiocyte lipoapoptosis in an animal model of NASH. Further, these results suggest that upregulation of miR-34a plays an important role in cholangiocyte lipoapoptosis in a cell culture model and potentially in biliary injury during NASH.

## DISCUSSION

Increased FFAs in the circulation during metabolic syndrome or NASH have fatal consequences in the liver by inducing cholangiocyte and hepatocyte lipoapoptosis (27, 28). Increased plasma FFAs have been observed in patients with NAFLD or the associated condition of metabolic syndrome. Plasma FFAs in 27 out of 47 NASH patients were elevated with a mean of 752 μM in NASH patients (29). De Almeida et al. (30) showed that total plasma FFAs were 350 μM in controls and 563 μM in NASH patients. Further, saturated FFA levels were 200 μM in controls and 316 μM in NASH patients. Control patients similarly had 460 μM FFA levels, compared with NASH patients at 660 μM (31). Recently, Zhang et al. (32) found that control patients had 450 μM plasma FFAs compared with 650 μM in NAFLD patients. Further, patients with evidence of advanced fibrosis had higher FFA levels (720 μM) than patients without evidence of advanced fibrosis (580 μM). From these studies, the range for controls was 350–460 μM FFAs, while patients had 563–752 μM circulating FFAs. These are total levels of all FFAs combined. In our study, we have utilized a specific saturated FFA, PA, to reduce complexity and investigate specific signaling pathways. We chose this fatty acid because it is the most common saturated FFA in the circulation [approximately 28% of the total FFAs (33), with a range of 300–4,100 μM in one study (34)]. Thus, our studies at 200–800 μM fall within the pathologic range of FFA concentrations.

**Fig. 8.**
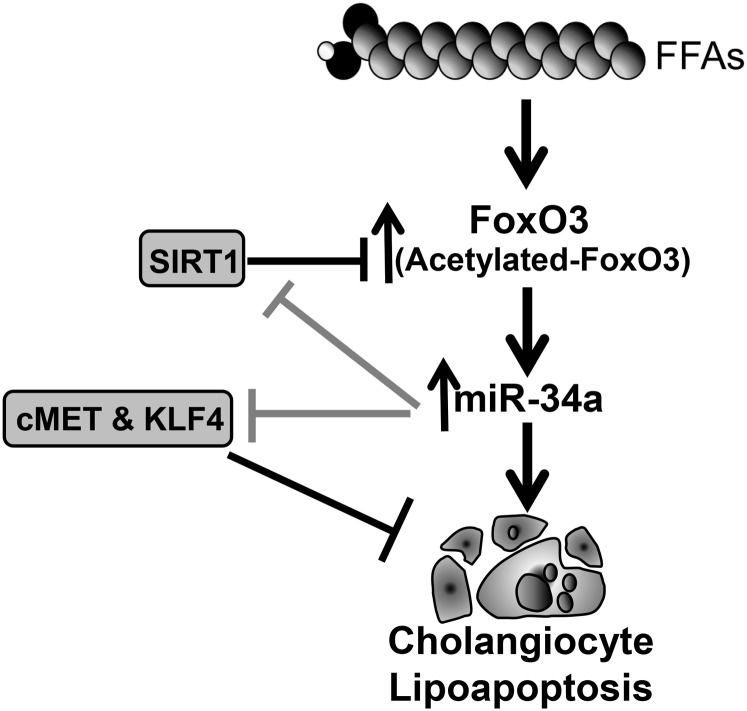
Schematic diagram of the proposed role of miR-34a in PA-induced cholangiocyte lipoapoptosis. The saturated FFA, PA, increased nuclear FoxO3 levels leading to increased miR-34a in PA-treated cells. miR-34a then promoted lipoapoptosis, likely through decreasing protein levels of its targets, SIRT1, MET, and KLF4. Based on literature reports of SIRT1 activity on FoxO3 and our previous finding of increased nuclear acetylated-FoxO3, we speculate that a miR-34a-mediated decrease in SIRT1 may enhance FoxO3 acetylation levels.

The principal finding of this study is that PA, the predominant circulating FFA, induced cholangiocyte lipoapoptosis via FoxO3-dependent induction of pro-apoptotic miR-34a. Our data suggest three important findings in cholangiocyte lipoapoptosis caused due to PA exposure: *1*) PA upregulates pro-apoptotic miR-34a in a FoxO3-dependent manner to cause cholangiocyte lipoapoptosis; *2*) increased miR-34a expression targets protein deacetylase SIRT1 as well as several anti-apoptotic proteins; and 3) cholangiocyte lipoapoptosis and miR-34a upregulation due to lipotoxicity were also evident in an animal model of NASH. The proposed model for FFA-induced cholangiocyte lipoapoptosis is shown in **Fig. 8**. These results will be discussed in the following paragraphs.

Biliary injury and cholestatic NASH have been documented at least in a subset of patients with NAFLD (3). Our earlier work showed implications for biliary injury in response to FFAs and demonstrated the mechanism of FFA-induced cholangiocyte lipoapoptosis involving the activation and nuclear translocation of FoxO3. Activation of FoxO3 with saturated FFAs thereby increased the expression of its downstream target, PUMA, a pro-apoptotic BH3-domain-containing protein, resulting in caspase-dependent cholangiocyte lipoapoptosis (4, 5). RNAi-mediated inhibition of PUMA protected cells partially, but not completely (5). Here, we extended our work to demonstrate the role for pro-apoptotic miR-34a in cholangiocyte lipoapoptosis. miR-34a has been shown to be upregulated in the liver from patients with NASH (15). Circulating levels of miR-34a were also shown to be elevated in patients with NAFLD, NASH, diabetes, obesity, and alcoholic liver disease (18, 25, 35). In the present study, we elucidated the mechanism of miR-34a upregulation. Our data also indicate that miR-34a upregulation with saturated FFA is a common process for both cholangiocyte- and hepatocyte-derived cells. Further, transfection of an antisense oligonucleotide against miR-34a prevented cholangiocyte lipoapoptosis due to PA exposure. Caspase 3 activation has been suggested to lead to cleavage of Dicer, a microRNA processing enzyme in the cytosol (36). However, we observed a dramatic increase in mature miR-34a levels with PA treatment in a panel of cholangiocytes tested, suggesting that Dicer is either not degraded in lipoapoptosis or it is not limiting for microRNA processing. We know that both cholangiocytes and hepatocytes are derived from a common progenitor cell. We observed that miR-34a upregulation was not solely occurring in cholangiocytes, but was also seen in Huh-7 cells. The increase in total hepatic miR-34a, either in our HFHS mice or in studies on NASH miRs (15), is likely to be dominated by hepatocyte-derived miR-34a, as hepatocytes comprise the majority of the cells in the liver. Whether miR-34a upregulation with FFAs in hepatocytes occurs in a similar FoxO3-dependent fashion, as in cholangiocytes, remains to be tested.

Our previous study implicated FoxO3 activation and nuclear translocation in cholangiocyte lipoapoptosis (5). We also reported that acetylated FoxO3 accumulated in the nuclear fraction in saturated FFA-treated cells (5). We speculated before that increased acetylation of FoxO3 might be due to a decrease in SIRT1 protein levels (5). Loss of SIRT1 has also been shown to be associated with insulin resistance (37, 38). Here we found that miR-34a targeted SIRT1 protein. SIRT1 is a protein deacetylase and acetylated FoxO3 is a known SIRT1 substrate (39, 40), so decreased SIRT1 can enhance the acetylation status of FoxO3. Acetylated FoxO3 was shown to be pro-apoptotic in nature and could induce downstream targets, like BIM and PUMA. Interestingly, we found that FoxO3 is responsible for the upregulation of miR-34a upon PA treatment. FoxO3 can increase Bcl-2 expression under normal conditions and in alcoholic liver disease, while acetylated-FoxO3 has been shown to have lost this protective function (41). Thus, the increased acetylated FoxO3 in cholangiocytes exposed to PA might have lost a Bcl-2-dependent protective function, instead becoming pro-apoptotic in nature.

Mature miR-34a has sequence complementarity to the 3′ UTR of *SIRT1* mRNA. NAFLD severity in human and animal models was shown to be positively correlated with the levels of miR-34a and inversely correlated with SIRT1 proteins levels (14). Treatment with ursodeoxycholic acid (UDCA) prevents the upregulation of miR-34a in total liver and primary hepatocytes (14). UDCA has been shown to upregulate the expression of SIRT1 to deacetylate p53, a miR-34a activator. Further, UDCA prevents posttranslational modification of p53 and reduces its transcriptional activity (14). Our results suggest that PA-induced miR-34a upregulation is independent of p53 transcriptional activity. Specifically, we observed a similar increase in miR-34a levels in both normal cholangiocytes and malignant-derived cholangiocytes that were confirmed by sequencing to have p53 inactivating mutants (data not shown). Using an inhibitor of SIRT1, we enhanced cytotoxicity of low-dose PA, demonstrating that loss of SIRT1 expression increased cell injury. SIRT1 deacetylase activity has been shown to have a role in the miR-34a promoter acetylation (9). Loss of SIRT1 results in failure of the miR-34a promoter to be deacetylated, which could possibly enhance FoxO3 transcriptional activation of miR-34a (9). Additionally, SIRT1 can deacetylate various other transcription factors, like FXR, SREBPs, PPARs, PGC1α, LXR, FoxO1, and FoxO3 (39, 42, 43). Deacetylation of FXR, LXR, PPARs, and PGC1α can activate their downstream targets with key roles in bile acid, cholesterol, lipid, and energy homeostasis (44). In contrast, deacetylation of SREBP can prevent its transcriptional activity (44). Whether these altered transcription factors have a role in cholangiocyte lipoapoptosis needs further investigation.

KLF4 is a zinc-finger transcription factor, tumor suppressor, and embryonic progenitor cell differentiation factor (45). KLF4 has been shown to act as a radio-protector against apoptotic insults (46). KLF4-deficient cells had decreased levels of the multi-domain Bcl-2 family proteins, Mcl-1 and Bcl-XL (BCL_XL_) (47). Further, KLF4 was shown to have transcriptional activity in the *MCL1* promoter region (47). PA-induced miR-34a decreased KLF4 levels in cholangiocyte-derived cells. Based on miR-34a regulation of KLF4 in the literature (6), KLF4 may be performing an anti-apoptotic role in cholangiocytes that is lost upon FFA treatment. We also observed that MET a receptor tyrosine kinase and hepatocyte growth factor receptor, is another anti-apoptotic protein targeted by miR-34a that was found to be decreased in cholangiocytes exposed to FFA. MET is well-known to activate Akt and Stat3 cell survival pathways (48). Additionally, the cell survival mechanism of MET also involves sequestering Fas to block apoptosis and prevent degradation of the anti-apoptotic regulator, FLIP_L_ (long splice form of FLICE inhibitor protein) (49). Normally, FLIP binds to Fas-associated death domain, a common protein for both Fas and death receptor complexes, and forms an apoptosis inhibitory complex that prevents the activation of pro-caspases (49). Further studies are needed to elucidate whether loss of KLF4 or MET is critical for cholangiocyte lipoapoptosis, but a MET inhibitor did not enhance killing.

Treatment with a miR-34a inhibitor has been suggested to be a novel therapeutic strategy for NAFLD (50). Silencing of miR-34a using a miR-34a inhibitor was shown to prevent liver injury and improve hepatic steatosis through its target proteins, like PPARα and SIRT1, which can activate AMP-activated protein kinase in an animal model of NAFLD (50). Additionally, FGFR signaling promotes cell survival (26), such that any loss of FGFR expression due to saturated FFA, through increased miR-34a, could promote lipoapoptosis. One of the downstream signaling pathways of FGFRs is Akt, and miR-34a targeting of FGFRs may decrease the levels of Akt that can affect the phosphorylation status of FoxO3 and AMP-activated protein kinase, as reported before (50). Ectopic miR-34a expression has been shown to decrease basal Akt phosphorylation and several proteins that regulate cell cycle that help in G1/S transition, like CKD4, CKD6, CCND1, CCNE2, and E2F3 (51). These observations further support our hypothesis that miR-34a is critical for cholangiocyte lipoapoptosis.

Our current and previous work demonstrates that saturated FFA induced cholangiocyte lipoapoptosis. We also observed that increased cholangiocyte lipoapoptosis was present in the nutrient-excess model for NASH. This model mimics a Western diet containing high saturated fats, cholesterol, and sucrose and shows similar characteristics to human NASH, such as ballooned hepatocytes, massive lipid droplet accumulation, increased collagen accumulation, increased α-smooth muscle actin expression, and increased pro-inflammatory cytokines in the liver. We observed enhanced levels of miR-34a in mice fed the HFHS diet, consistent with a role for miR-34a in the lipoapoptosis of NASH. A recent study showed that lentiviral injection of miR-34a inhibitor resulted in protection against liver injury (50). We predict that treatment with a miR-34a inhibitor might also prevent cholangiocyte lipoapoptosis. Further studies are required to elucidate whether cholangiocyte lipoapoptosis contributes to or promotes the progression of NASH, at least in a subset of cholestatic NAFLD patients.

In conclusion, our results show that PA-induced cholangiocyte lipoapoptosis involved the activation of pro-apoptotic miR-34a, dependent on FoxO3. miR-34a targeted protein deacetylase, SIRT1, and anti-apoptotic proteins. Further, cholangiocyte lipoapoptosis was also evident in an animal model of NASH.
